# Synthesis of Isomeric and Potent Impurities of the Triazole-Based Antifungal Drug Voriconazole

**DOI:** 10.3797/scipharm.1501-13

**Published:** 2015-03-06

**Authors:** Dhanraj T. S. S. Sundaram, Jayati Mitra, Aminul Islam, Koilpillai Joseph Prabahar, Battula Venkateswara Rao, Sanasi Paul Douglas

**Affiliations:** 1 Chemical Research and Development, APL Research Centre-II, Aurobindo Pharma Ltd., Survey No. 71 & 72, Indrakaran (V), Medak Dist.-502329, Andhra Pradesh, India; 2 Department of Engineering chemistry, Andhra University College of Engineering (A), Andhra University, Visakhapatnam-530003, Andhra Pradesh, India

**Keywords:** Voriconazole, Impurity synthesis, Positional isomers, Chloroacetyl chloride

## Abstract

We describe the synthesis of two positional isomers and a desfluoro impurity of Voriconazole starting with Friedel–Crafts acylation of mono- and difluorobenzene. These isomers are the crucial components in determining the quality of Voriconazole during its manufacturing from the key raw material, 1-(2,4-difluorophenyl)-2-(1*H*-1,2,4-triazol-1-yl)ethan-1-one. All the prepared impurities were characterized by IR, ^1^H-NMR, ^13^C-NMR, and mass spectral data.

## Introduction

Voriconazole **1** is a triazole antifungal medication used to treat serious fungal infections in patients who are immunocompromised [1]. This includes invasive candidiasis, invasive aspergillosis, and emerging fungal infections. It is chemically known as (*2R,3S*)-2-(2,4-difluorophenyl)-3-(5-fluoropyrimidin-4-yl)-1-(1*H*-1,2,4-triazol-1-yl)butan-2-ol.

**Fig. 1:**
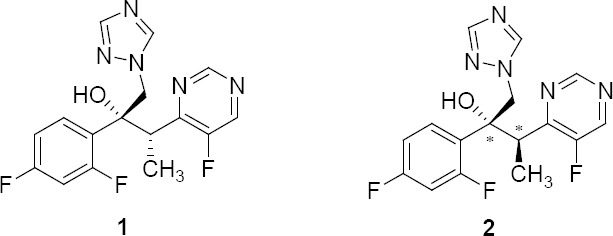
Structure of Voriconazole **1** (2R,3S) and its diastereomer **2** (2R,3R/2S,3S)

It is generally seen that during the manufacturing of APIs (Active Pharmaceutical Ingredient), some of the related impurities (especially the isomeric) are retained in the final API and may have an impact on the quality and safety of the drug substance based on its toxicology. Therefore, it is important to study the origin of such impurities followed by their control and elimination. Furthermore, it is equally important for synthesis to identify and characterize such impurities in a pure form to check the analytical performance characteristics such as specificity, linearity, accuracy, limit of detection (LOD), limit of quantification (LOQ), robustness, and relative retention factor [2]. It is recommended by the International Conference on Harmonization (ICH) guidelines that the impurities which are present in the API at a level ≥ 0.05% w/w should be identified and characterized [3].

During the process development of **1** in our laboratory, we have observed the presence of an isomeric mass relative to **1** in the crude product from the LC-MS studies. In our present work, we have demonstrated the preparation of these impurities for the better understanding of the impurity profiling of **1**. As these isomeric impurities may have the same characteristic properties as do the product, we have focused our attention towards its control and elimination. We presumed that these impurities may have increased due to the contamination of **3** (1-(2,4-difluorophenyl)-2-(1*H*-1,2,4-triazol-1-yl)ethan-1-one) containing differently substituted fluorobenzene.

## Results and Discussion

To the best of our knowledge, there are two major routes described in the literature for the preparation of **1**, as shown in Scheme 1 [4–6]. In the case of route A, deprotonation of 4-chloro-6-ethyl-5-fluoropyrimidine (**4**) was carried out using LDA as a base in THF at very low temperature and treated with ketone **3** to obtain the alcohol adducts **5** and **6**. The handling of LDA at low temperature, poor stability of the lithium anion of **4**, and enolisation of **3** in the presence of lithium carbanions were the major issues in this route. In addition to this, poor diastereoselectivity (~1:1 mixture of **5** and **6**) and low product conversion (~20%) has led us to an alternative route. In route B, ketone **3** was treated with 6-(1-bromoethyl)-2-chloro-5-fluoropyrimidine (**8**) in the presence of activated zinc dust containing a catalytic amount of lead powder. The product conversion observed in this route was far better than the earlier route with excellent diastereoselectivity. The ratio of diastereomeric pairs **5** and **6** were ~1:0.14 in the reaction mixture with more than 60% product conversion. The unwanted diastereomeric pair **6**, which was formed in a minor quantity, was eliminated during crystallization in the next stages. Based on the diastereoselectivity of the process, we have prepared the impurities by following route B.

**Sch. 1:**
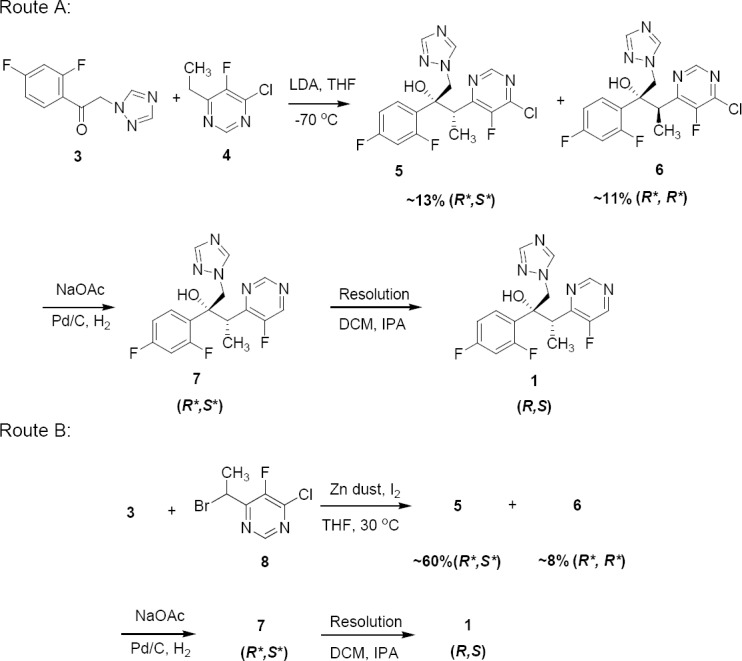
Reported synthesis of Voriconazole 1 (Route A and Route B)

The key raw material **3** was prepared by the Friedel–Crafts acetylation of 1,3-difluorobenzene with chloroacetyl chloride (Scheme 2). It is believed that during the preparation of 1,3-difluorobenzene from fluorobenzene, other positional isomers of disubstituted fluorobenzene such as 1,2-difluorobenzene and 1,4-difluorobenzene can form and may carry due to their closer boiling points (82–92°C) with fluorobenzene [7]. The ketones **3a–c,** prepared using 1,2-difluorobenzene, 1,4-difluorobenzene, and fluorobenzene were treated with an organozinc derivative of **8** to obtain an enantiomeric pair of **5a–c** (Scheme 3). Further dechlorination of **5a–c** under catalytic hydrogenation in the presence of sodium acetate led to the formation of an enantiomeric pair **7a–c**.

**Sch. 2:**
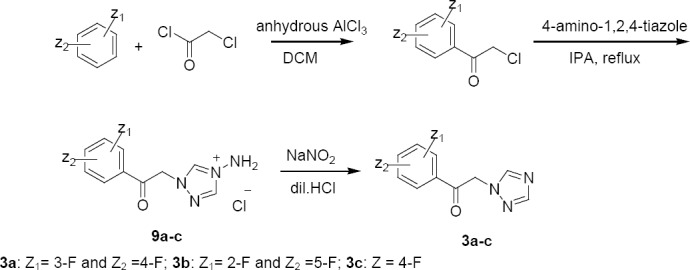
Synthesis of mono- and (difluorophenacyl)triazole **3a–c**

**Sch. 3:**
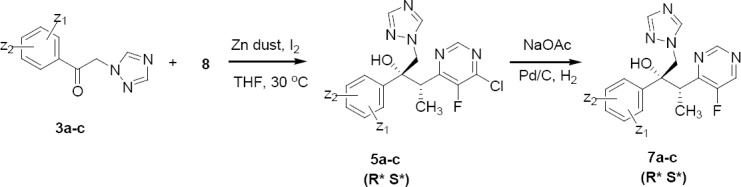
Synthesis of **7a–c**

The -*CH_3_* protons in ^1^H-NMR for **5** appeared at δ 1.14 *ppm* (*J* = 9.0 Hz), whereas for **6** a downfield shift was observed with a δ value of 1.49 *ppm* (*J* = 6.6 Hz). The δ value corresponding to **5a–c** is very close to **5** which appeared at ~ 1.14–1.17 *ppm* (*J* = 7–7.5 Hz). As the preparation of **7a–c** was similar to **1**, its stereochemistry was confirmed by the relative studies from **1** and **2**. The signal corresponding to the -C*H_3_* protons of **1** appeared at δ 1.14 *ppm* (*J* = 6.9 Hz), whereas for the diastereomeric pair **2**, it appeared at δ 1.49 *ppm* (*J* = 6.9 Hz). In the case of **7a–c**, we have observed the signals for -C*H_3_* protons in the range nearby δ 1.14–1.16 *ppm*, which assured us that the formed compounds were the enantiomeric pairs (R* S*) and not the diastereomeric pairs (R* R*). The stereochemistry was further confirmed by the chemical shift values from the ^13^C-NMR of **1** (δ = 13.8 *ppm*) with **7a–c** (δ = 13.8) of the -*C*H*_3_* carbon atom.

**Fig. 2:**
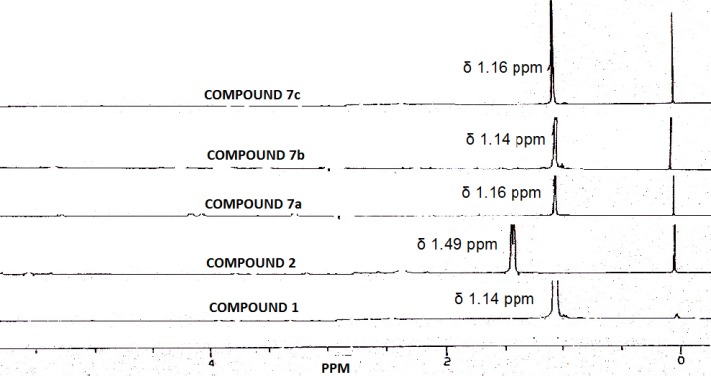
Comparative chemical shift values of –CH_3_ protons in ^1^H-NMR of compound 1, 2, and **7a–c**

## Conclusion

The plausible impurities of Voriconazole, starting from mono- and difluorobenzene were prepared and clearly identified by spectral techniques based on the comparative studies against **1** and **2.**

## Experimental

Melting points were determined with a Reichert Thermopan apparatus. The ^1^H- and ^13^C-NMR spectra were recorded with a Bruker Avance 300 MHz and Varian 500 MHz spectrometer using TMS as the internal standard in DMSO-*d*_6_ or CDCl_3_. The IR spectra were recorded using a Perkin-Elmer Spectrum One Fourier Transform (FTIR) spectrophotometer. High-resolution mass spectral analyses were performed using the electrospray ionization (ESI) method on the Xevo G2 QTOf mass spectrometer. HPLC measurements were run on a Novapak C_18_ column (150 mm x 3.9 mm, 4 µm) with a mobile phase containing buffer (pH = 4), methanol, and acetonitrile in a ratio of 550:300:150 v/v/v. The flow rate was maintained at 1.0 mL/min with a column temperature of 35°C. UV detection occurred at λ = 256 nm. All reagents were used as purchased unless otherwise stated. Reactions were performed under a nitrogen atmosphere and the work-ups were done in a well-ventilated fuming hood.

### General Procedure for the Preparation of 3a–c

To a stirred solution of mono- or difluorophenacyl chloride (24.60 g, 0.14 mol) in 2-propanol (50 mL), 4-amino-4*H*-1,2,4-triazole (10.0 g, 0.12 mol) was added. The suspension was stirred at reflux for 6 h, cooled to 25–30°C and stirred for another 1 h at 25–30°C. The solid was filtered and washed with 2-propanol (10 mL) and dried under reduced pressure (10–15 mm Hg) for 6 h at 45°C to yield **9a–c** in good yield. The crude product **9a–c** (20.0 g, 0.08 mol) thus obtained was dissolved in 1 N HCl (100 mL) at 2–5°C. To the above, an aqueous solution of sodium nitrite (6.46 g, 0.09 mol, 40 mL) was slowly added over a period of 30 min at 2–5°C under stirring. The reaction mixture was stirred for 1 h at 2–5°C, and later the temperature was raised to 25–30°C and stirred for another 1 h at 25–30°C. The solid obtained was filtered, washed with water (20 mL), and dried under reduced pressure (10–15 mm Hg) at 50°C for 8 h to obtain **3a–c**.

#### 1-(3,4-Difluorophenyl)-2-(1H-1,2,4-triazol-1-yl)ethan-1-one (3a)

Brown solid; Yield 84.1%; mp 120–122°C; IR (KBr) 3058, 2982, 1704, 1607, 1512, 1462, 1434, 1142, 842, 681 cm^−1^; ^1^H-NMR (500 MHz, DMSO-d_6_) δ 6.00 (s, 2H), 7.67–7.73 (m, 1H), 7.95–7.98 (br s, 1H), 8.04 (s, 1H), 8.11–8.15 (m, 1H), 8.51 (s, 1H); HRMS (ESI, QTOF) for C_10_H_7_F_2_N_3_O [M+H]^+^: *m/z* calcd: 224.0557; found: 224.0653.

#### 1-(2,5-Difluorophenyl)-2-(1H-1,2,4-triazol-1-yl)ethan-1-one (3b)

Yellow solid; Yield 81.5%; mp 124–126°C; IR (KBr) 3055, 2981, 1707, 1621, 1513, 1489, 1419, 1258, 1138, 839, 680 cm^−1^; ^1^H-NMR (500 MHz, CDCl_3_) δ 5.61 (s, 2H), 7.21–7.24 (m, 1H), 7.33–7.36 (m, 2H), 8.02 (s, 1H), 8.22 (s, 1H); HRMS (ESI, QTOF) for C_10_H_7_F_2_N_3_O [M]^+^: *m/z* calcd: 224.0557; found: 224.0655.

#### 1-(4-Fluorophenyl)-2-(1H-1,2,4-triazol-1-yl)ethan-1-one (3c)

Light brown solid; Yield 76.5%; mp 106–108°C; IR (KBr) 3054, 2981, 1689, 1599, 1450, 1415, 1271, 1142, 839, 677 cm^−1^; ^1^H-NMR (500 MHz, CDCl_3_) δ 5.65 (s, 2H), 7.21-7.26 (m, 2H), 8.01–8.05 (m, 3H), 8.25 (s, 1H); HRMS (ESI, QTOF) for C_10_H_8_FN_3_O [M+H]^+^: *m/z* calcd: 206.0651; found: 206.0745.

### General Procedure for the Preparation of 5a–c

To a stirred suspension of zinc dust (7.90 g, 0.12 mol) and lead powder (5% w/w) in THF (50 mL), a solution of iodine (6.20 g, 0.02 mol) in THF (15 mL) was added over a period of 30 min. The suspension was stirred at 25°C for 30 min and further cooled to 8–10°C. Thereafter, a solution of **3a–c** (5.0 g, 0.02 mol), **8** (7.60 g, 0.03 mol), and iodine (1.0 g, 0.004 mol) in THF was added within 20 min at 8–25°C. The reaction mixture was stirred at 25°C for 2 h. The reaction mixture was filtered to remove the salts and acetic acid (7.20 g, 0.12 mol) was added. The pH was adjusted to 8.5 using 30% w/w aqueous sodium carbonate solution under stirring. The suspension was filtered after 30 min to remove the inorganic solid particles. The filtrate was concentrated under reduced pressure to remove THF. The product was extracted with toluene (2 x 50 mL). The organic layer was concentrated and the product was isolated using ether (20 mL) to obtain **5a–c**.

#### rel-(2R,3S)-3-(6-Chloro-5-fluoropyrimidin-4-yl)-2-(3,4-difluorophenyl)-1-(1H-1,2,4-triazol-1-yl)butan-2-ol (5a)

Pale yellow solid; Yield 58.8%; mp 129–131°C; Purity 98.89%; IR (KBr) 3260, 3139, 3110, 3080, 1572, 1508, 1396, 1034, 820, 744, 696 cm^−1^; ^1^H-NMR (500 MHz, DMSO-d_6_) δ 1.17 (d, 3H, *J* = 7.5 Hz), 3.71-3.74 (m, 1H), 4.69-4.76 (ABq, 2H, *J* = 15.0 Hz), 5.92 (s, 1H), 7.12–7.14 (m, 1H), 7.29–7.39 (m, 2H), 7.80 (s, 1H), 8.26 (s, 1H), 8.77 (s, 1H); HRMS (ESI, QTOF) for C_16_H_13_Cl F_3_N_5_O [M+H]^+^: *m/z* calcd: 384.0840; found: 384.0834.

#### rel-(2R,3S)-3-(6-Chloro-5-fluoropyrimidin-4-yl)-2-(2,5-difluorophenyl)-1-(1H-1,2,4-triazol-1-yl)butan-2-ol (5b)

Beige solid; Yield 44.7%; mp 106–108°C; Purity 93.68%; IR (KBr) 3316, 3116, 3107, 3082, 1566, 1482, 1393, 1033, 812, 774, 697, 683 cm^−1^; ^1^H-NMR (500 MHz, DMSO-d_6_) δ 1.14 (d, 3H, *J* = 6 Hz), 3.96–3.99 (m, 1H), 4.41–4.44 (d, 1H, *J*=14.5Hz), 4.38–4.82 (d, 1H), 5.99 (s, 1H), 6.96–7.00 (m, 1H), 7.13–7.18 (m, 1H), 7.21–7.26 (m, 1H), 7.64 (s, 1H), 8.23 (s, 1H), 8.90 (s, 1H); HRMS (ESI, QTOF) for C_16_H_13_Cl F_3_N_5_O [M+H]^+^: *m/z* calcd: 384.0840; found: 383.9809.

#### rel-(2R,3S)-3-(6-Chloro-5-fluoropyrimidin-4-yl)-2-(4-fluorophenyl)-1-(1H-1,2,4-triazol-1-yl)butan-2-ol (5c)

Brown solid; Yield 48.7%; mp 138–140°C; Purity 96.84%; IR (KBr) 3233, 3146, 3114, 3088, 1573, 1508, 1397, 1034, 812, 757, 693cm^−1^; ^1^H-NMR (500 MHz, DMSO-d_6_) δ 1.17 (d, 3H, *J* = 7.0 Hz), 3.68–3.69 (m, 1H), 4.68–4.77 (ABq, 2H), 5.84 (s, 1H), 7.05–7.09 (dd, 2H, *J* = 7.5 Hz), 7.29–7.32 (dd,1H, *J* = 5.5 Hz), 7.80 (s, 1H), 8.24 (s, 1H), 8.75 (s, 1H); HRMS (ESI, QTOF) for C_16_H_14_Cl F_2_N_5_O [M+H]^+^: *m/z* calcd: 366.0934; found: 366.0930.

### General Procedure for the Preparation of 7a–c

To a stirred solution of **5a–c** (4.0 g, 0.01 mol) in ethanol (20 mL), sodium acetate (0.86 g, 0.01 mol) was added. Thereafter, 10% palladium on carbon (0.2 g) was added and stirred in an autoclave under hydrogen pressure of 4–6 kg/cm^2^ for 3 h at 25–30°C. The reaction mixture was filtered and concentrated under reduced pressure at 45°C. To the residue, 20% v/v aqueous ethanol (20 mL) was added and stirred for 1 h at 25–30°C. The precipitated solid was filtered, washed with water (10 mL), and dried under reduced pressure to obtain **7a–c**.

#### rel-(2R,3S)-2-(3,4-Difluorophenyl)-3-(5-fluoropyrimidin-4-yl)-1-(1H-1,2,4-triazol-1-yl)butan-2-ol (7a)

White solid; Yield 82.4%; mp 142–144°C; Purity 99.48%; IR (KBr) 3390, 3116, 3086, 1588, 1461, 1409, 1379, 1058, 822, 779, 710, 680 cm^−1^; ^1^H-NMR (500 MHz, DMSO-d_6_) δ 1.16 (d, 3H, *J* = 7.5 Hz), 3.71–3.74 (q, 1H), 4.63–4.77 (ABq, 2H), 6.00 (s, 1H), 7.11–7.13 (m, 1H), 7.28–7.36 (m, 2H), 7.76 (s, 1H), 8.25 (s, 1H), 8.78 (d, 1H, *J* = 2.0 Hz), 8.95 (d, 1H, *J* = 2.0 Hz); ^13^C-NMR (125 MHz, DMSO-d_6_, PENDANT) δ 13.9, 40.4, 55.8, 78.6, 115.6, 116.4, 123.2, 139.8, 144.8, 146.4, 149.5, 150.5, 153.6, 154.2, 157.3, 157.9 *ppm*; HRMS (ESI, QTOF) for C_16_H_14_F_3_N_5_O [M+H]^+^: *m/z* calcd: 350.1229; found: 350.1223.

#### rel-(2R,3S)-2-(2,5-Difluorophenyl)-3-(5-fluoropyrimidin-4-yl)-1-(1H-1,2,4-triazol-1-yl)butan-2-ol (7b)

Off-white solid; Yield 78.9%; mp 130–132°C; Purity 93.49%; IR (KBr) 3424, 3140, 3080, 1584, 1483, 1405, 1029, 813, 769, 708, 677 cm^−1^; ^1^H-NMR (500 MHz, DMSO-d_6_) δ 1.14 (d, 3H, *J* = 7.5 Hz), 3.96–4.00 (m, 1H), 4.32–4.36 (d, 1H, *J* =14.5 Hz), 4.81–4.85 (d, 1H), 6.06 (s, 1H), 6.97–7.01 (m, 1H), 7.13–7.17 (m, 1H), 7.20–7.25 (m, 1H), 7.62 (s, 1H), 8.25 (s, 1H), 8.78 (d, 1H, *J* =2.5Hz), 9.05 (d, 1H, *J* =2.5 Hz); ^13^C-NMR (125 MHz, DMSO-d_6_, PENDANT) δ 13.7, 39.3, 54.9, 77.4, 115.9, 117.4, 130.1, 144.8, 145.2, 150.4, 152.1, 153.9, 154.6, 157.2, 158.2, 159.8 *ppm*; HRMS (ESI, QTOF) for C_10_H_14_F_3_N_5_O [M+H]^+^: *m/z* calcd: 350.1229; found: 350.0291.

#### rel-(2R,3S)-2-(4-Fluorophenyl)-3-(5-fluoropyrimidin-4-yl)-1-(1H-1,2,4-triazol-1-yl)butan-2-ol (7c)

Pale yellow solid; Yield 86.5%; mp 160–162°C; Purity 99.65%; IR (KBr) 3400, 3135, 3067, 1588, 1461, 1408, 1378, 1058, 816, 778, 709, 679 cm^−1^; ^1^H-NMR (500 MHz, DMSO-d_6_) δ 1.16 (d, 3H, *J* = 7.5 Hz), 3.69 (m, 1H), 4.64–4.77 (ABq, 2H, *J* =14 Hz), 5.91 (s, 1H), 7.04-7.07 (m, 2H), 7.29–7.32 (m, 2H), 7.76 (s, 1H), 8.23 (s, 1H), 8.75 (d, 1H, *J* = 2.5 Hz), 8.93 (d, 1H, *J* = 2.5Hz); ^13^C-NMR (125 MHz, DMSO-d_6_, PENDANT) δ 13.9, 40.6, 56.1, 77.2, 114.3, 128.2, 136.8, 144.4, 144.9, 150.4, 153.5, 154.8, 156.5, 156.7, 159.2, 163.4 *ppm*; HRMS (ESI, QTOF) for C_16_H_15_F_2_N_5_O [M+H]^+^: *m/z* calcd: 332.1324; found: 332.0439.
